# VIPE: Visible and Infrared Fused Pose Estimation Framework for Space Noncooperative Objects

**DOI:** 10.3390/s25216664

**Published:** 2025-11-01

**Authors:** Zhao Zhang, Dong Zhou, Yuhui Hu, Weizhao Ma, Guanghui Sun, Yuekan Zhang

**Affiliations:** 1 Department of Control Science and Engineering, Harbin Institute of Technology, Harbin 150001, China; zhangzhao97@hit.edu.cn (Z.Z.); huyuhui@hit.edu.cn (Y.H.); 24s104150@stu.hit.edu.cn (W.M.); guanghuisun@hit.edu.cn (G.S.); 2 College of Mechanical & Electronic Engineering, Shandong University of Science and Technology, Qingdao 266590, China; zhangyk2007@sdust.edu.cn

**Keywords:** visible-infrared fusion, pose estimation, space non-cooperative objects, deep-learning

## Abstract

Accurate pose estimation of non-cooperative space objects is crucial for applications such as satellite maintenance, space debris removal, and on-orbit assembly. However, monocular pose estimation methods face significant challenges in environments with limited visibility. Different from the traditional pose estimation methods that use images from a single band as input, we propose a novel deep learning-based pose estimation framework for non-cooperative space objects by fusing visible and infrared images. First, we introduce an image fusion subnetwork that integrates multi-scale features from visible and infrared images into a unified embedding space, preserving the detailed features of visible images and the intensity information of infrared images. Subsequently, we design a robust pose estimation subnetwork that leverages the rich information from the fused images to achieve accurate pose estimation. By combining these two subnetworks, we construct the Visible and Infrared Fused Pose Estimation Framework (VIPE) for non-cooperative space objects. Additionally, we present a Bimodal-Vision Pose Estimation (BVPE) dataset, comprising 3,630 visible-infrared image pairs, to facilitate research in this domain. Extensive experiments on the BVPE dataset demonstrate that VIPE significantly outperforms existing monocular pose estimation methods, particularly in complex space environments, providing more reliable and accurate pose estimation results.

## 1. Introduction

Proximity operations in on-orbit servicing, such as debris removal [1,2] and the repair of malfunctioning spacecraft [3,4,5,6], critically depend on the accurate pose estimation of non-cooperative space objects. This capability ensures precise alignment, manipulation, and interaction with target objects, playing a pivotal role in mission success and operational safety.

Compared to vision-based sensors, radar systems exhibit significantly higher mass and lower energy efficiency, rendering them less suitable for space applications. Depth sensors, on the other hand, are constrained by their limited measurement range and dynamic performance, making them generally unsuitable for non-indoor environments. While stereoscopic cameras can provide depth information, they require a large baseline to achieve acceptable accuracy, which increases system complexity and reduces the field of view. In contrast, vision-based pose estimation has gained increasing popularity due to its advantages in power efficiency, reduced hardware complexity, cost-effectiveness, and superior dynamic performance [7].

Space objects can be categorized as cooperative or non-cooperative based on their communication capabilities and the presence of distinctive markings [8]. Cooperative objects can actively transmit pose information via radio communication. In contrast, non-cooperative objects lack these capabilities, rendering pose estimation significantly more challenging [9,10].

Vision-based pose estimation for non-cooperative spacecraft has been a subject of extensive research for many years. Early approaches [11,12,13,14] primarily relied on traditional image processing techniques to extract handcrafted feature points, which were then matched with corresponding points on the target model to estimate the pose. Traditional image processing methods, such as SIFT, SURF, and ORB, are effective only for targets with strong textures [15,16]. However, they often struggle with targets exhibiting weak or no textures due to their reliance on feature points. For instance, smooth surfaces like ceramics or low-light conditions can lead to insufficient or unreliable keypoint detection, resulting in failed pose estimation. These limitations highlight the need for more robust approaches in texture-challenged scenarios. Additionally, their robustness and generalization capabilities are limited, making them susceptible to adverse lighting conditions and the dynamic backgrounds typical of space environments.

With the remarkable performance of deep learning in various computer vision tasks, deep convolutional neural networks (CNNs) have increasingly been employed for monocular image-based spacecraft pose estimation for known target [17]. Several CNN models have been developed to estimate the pose of spacecraft, which can be categorized into single stage methods and multi-stage methods based on their approach to pose estimation. Single stage methods attempt to learn the nonlinear mapping between input images and output poses, enabling end-to-end pose estimation of the spacecraft. In contrast, multi-stage methods first employ CNN-based keypoint detectors to predict the positions of 2D keypoints. Subsequently, they employ Perspective-n-Point (PnP) solvers to estimate the spacecraft pose based on the corresponding 3D coordinates of these keypoints. These two methods usually rely on single-band images (visible images). In the on-orbit scenario, non-cooperative objects predominantly undergo rotational motion, where conventional visible-image pose estimation faces significant challenges due to shadow occlusion, illumination fluctuations, and sensor noise, resulting in degraded accuracy. By contrast, infrared imaging demonstrates superior capability in capturing both structural contours and thermal signatures of space objects through solar thermal radiation detection. The proposed multimodal fusion approach effectively combines complementary information from different spectral bands, thereby establishing a more robust and reliable framework for space target pose estimation under various orbital conditions.

To address these challenges, we propose the Visible and Infrared Fused Pose Estimation Framework (VIPE) for space non-cooperative objects. VIPE leverages the additional intensity and thermal information provided by infrared images to enhance the robustness and accuracy of pose estimation in adverse space environments. VIPE mainly consists of image fusion sub-network and pose estimation subnetwork, which fully integrates the advantages of visible and infrared images to quickly and accurately estimate the 6-DOF (6-degrees of freedom) information of non-cooperative objects. Firstly, multi-scale convolutional neural network and Efficient Channel Attention (ECA) [18] were used to construct image fusion sub-networks to achieve feature extraction and feature level fusion of multi-source images. Then, we design a pose estimation network based on soft classification, where intuitive Euler angle labels are encoded into distinct spatial bins, facilitating end-to-end 6-DOF pose estimation.

A significant challenge addressed in this study is the lack of multi-source image datasets for pose estimation of space non-cooperative objects. Existing datasets, such as SPEED [19] and SPEED+ [20], are limited to visible images collected in simulated environments. To address this gap, we designed a non-cooperative object model and constructed a ground-based simulation experiment platform. By calibrating the platform in advance, we autonomously obtained accurate ground-truth pose data for the object. As a result, we introduce the Bimodal-Vision Pose Estimation (BVPE) dataset, which contains paired visible and infrared images for space non-cooperative objects. We evaluated the pose estimation accuracy of existing methods on the BVPE dataset, and the results demonstrate that VIPE exhibits superior robustness and effectively adapts to complex space environments. The BVPE dataset has significant theoretical and practical value with diverse potential applications. It can simulate on-orbit service tasks like satellite capture and maintenance, support space debris monitoring and removal, aid deep-space exploration, enhance space situational awareness through multi-target recognition, and collision avoidance.

Our contributions in this paper are summarized as follows:

- In this paper, a multi-scale feature image fusion subnetwork is designed, and a soft classification network is introduced to achieve end-to-end pose estimation. On this basis, we propose the Visible and Infrared Fused Pose Estimation Framework for Space Noncooperative Objects (VIPE).
- To the best of our knowledge, we present the first bimodal-vision pose estimation (BVPE) dataset for space noncooperative objects, which includes spacecraft model with varying poses that can be used to train and test networks.
- We conducted extensive experiments and comparisons between VIPE and other advanced pose estimation methods on the BVPE dataset. The results demonstrate that our approach significantly outperforms traditional methods that rely solely on a single type of imagery.

The rest of this paper is ordered in the subsequent sections. In Section 2, we introduce the work related to the pose estimation of space noncooperative objects. We present in detail the Visible and Infrared Fused Pose Estimation Framework (VIPE) for Space Noncooperative Objects in Section 3. In Section 4, we introduce the BVPE dataset, based on which we conduct experiments analyze the results. Finally, we make a conclusion in Section 5.

## 2. Related Work

In this section, we review existing research on monocular image-based pose estimation for non-cooperative spacecraft, which can be broadly classified into two categories: traditional methods and deep learning-based methods. Additionally, we provide a concise overview of existing algorithms for visible and infrared image fusion.

### 2.1. Traditional Methods

Traditional monocular vision-based pose estimation methods primarily depend on handcrafted features and prior geometric model knowledge of specific spacecraft. These methods employ traditional feature detection techniques, such as SIFT [21] and ORB [22], to extract features from images. The extracted features are then matched with predefined features of the known spacecraft, establishing correspondences between 2D keypoints and 3D model points to estimate the pose.

D’Amico et al. [11] used the Canny edge detector to extract edge features from images and determine the pose of the target spacecraft through model matching. Liu [12] utilized elliptical information to calculate the spacecraft orientation and distance parameters, leveraging the asymmetry of these features to ensure the accuracy of the pose estimation. Despite the effectiveness of these methods, their reliance on individual model characteristics does not guarantee robustness in pose estimation. To address these limitations, Sharma et al. [13] proposed weak gradient elimination (WGE), where the Sobel operator and Hough transform were constrained by geometric features to extract multiple image features, thereby improving the accuracy of pose estimation.

However, traditional pose estimation methods often require specific environmental conditions, such as good lighting and clear image quality. In complicated and changeable environments, such as those with strong light or shadows, the accuracy of these methods is limited by the precision of the model and the matching algorithm.

### 2.2. Deep Learning Based Methods

With the development of deep learning technology, many pose estimation methods based on deep learning have emerged in recent years. Deep learning-based approaches can be categorized into single stage and multi-stage methods, depending on whether the pose of the non-cooperative object is directly obtained.

The multi-stage method primarily relies on the geometric feature key points of the non-cooperative object. A neural network-based image key point regression model is designed to predict the 2D bounding box and the pixel positions of the 2D key points of the non-cooperative object. Subsequently, the relationship between the 2D key points and their corresponding 3D key points is calculated using PnP or EPnP solvers to obtain the 6-DOF pose of the camera relative to the object. Park [23] was the first to use an object detection network for ROI (Region of Interest) detection to determine the position of a spacecraft in an image, then returned the coordinates of the vector, and finally used the PnP method to solve for the pose. Similarly, Chen et al. [24] first detected the two-dimensional bounding box of the object, used HRNet [25] and heat maps to obtain key points, and then determined the relative orientation of the object and camera through the RANSAC PnP algorithm. Lorenzo et al. [26] combined a CEPPnP solver with an extended Kalman filter and a depth network, using heat maps to predict the two-dimensional pixel coordinates of feature points for non-cooperative spacecraft. Huo et al. [27] replaced the prediction head of tiny-YOLOv3 with key point detection results and designed a reliability judgment model and a key point existence judgment model to enhance the robustness of the pose estimation algorithm. The lightweight feature extraction network also facilitates deployment in space environments. Wang et al. [28] designed a key point detection network based on the Transformer model and introduced a representation containing key point coordinates and index entries. The final relative position of the camera and object was determined using the PnP method. Liu et al. [29] proposed a deformable-transformer-based single stage end-to-end SpaceNet (DTSE-SpaceNet) that integrates object detection with key point regression. Yu et al. [30] adopted a wire-frame-based landmark description method, performing direct coordinate classification for satellite landmark positioning, and finally used the PnP method to obtain the target pose. It would be more physically interpretable and theoretically robust to detect keypoints and apply a 2D-3D correspondence with a PnP solver.

Although based on the deep study of the multi-stage pose estimation method shows the potential, but almost all of the training images are synthetic. In practical space control missions, spacecraft are mostly non-cooperative objects, the key point regression used by the multi-stage approach may fail. The single stage method directly estimates the pose of the target from the input image through a model. The nonlinear mapping relationship between the image and the pose is typically established via a neural network through end-to-end training, which can simplify the processing pipeline and enhance real-time performance. Additionally, the single stage method does not require additional information such as the 3D model of the spacecraft and the corresponding 2D key points, making it more suitable for deployment in the real space environment. However, the single stage method requires the same camera parameters during training and testing.

Sharma et al. [31] use AlexNet to classify the input images with discrete orientation labels and transform the regression problem of orientation estimation into a classification problem. However, this method requires the orientation space to be discretized to a very detailed extent to ensure the accuracy of orientation estimation and it is limited by the number of training data samples. To address this issue, Proença and Gao [32] proposed a deep learning framework for spacecraft orientation estimation based on soft classification. This framework directly obtained the relative position through a regression method and the relative orientation through probabilistic direction soft classification; additionally, they proposed the URSO dataset. Garcia et al. [33] proposed a two-branch spacecraft pose estimation network. The first branch outputs the object position, and the second branch uses ROI images to return the spacecraft orientation quaternion. Huang et al. [34] proposed a pose estimation network with three branches. The first branch returns the quaternion information of the object through soft classification coding, the second branch returns the 3-DOF position information, and the third branch determines whether the results of the first two branches need to be output. Sharma et al. [35] proposed the Spacecraft Pose Network (SPN), which consists of three branch networks. These networks realize spacecraft position estimation, probability mass function prediction after discrete soft classification, and final orientation regression, respectively. The relative position of the spacecraft is obtained using the Gauss-Newton algorithm, and the target pose can be estimated using only grayscale images. To solve the problem of domain differences, Park et al. [36] improved upon SPN and proposed a new multi-task network architecture SPNv2.

The single stage pose estimation method exhibits lower accuracy compared to multi-stage approaches. However, it offers distinct advantages, including reduced dependency on additional labels, enhanced adaptability to complex environments through end-to-end training, and minimized reliance on data preprocessing. Existing pose estimation methods, whether single-stage or multi-stage, predominantly rely on visible images as network inputs. While effective in terrestrial applications, this approach presents significant limitations in space environments. Visible sensors often fail to capture object details under low-light or high-contrast conditions, while infrared sensors struggle in scenarios with minimal thermal gradients or high reflectivity. To address these challenges, we propose the Visible and Infrared Fused Pose Estimation Framework (VIPE) for space non-cooperative objects. VIPE leverages complementary features from both visible and infrared spectra through a novel fusion mechanism, providing richer and more robust input representations. This fusion not only overcomes the limitations of single-modal approaches but also significantly enhances pose estimation accuracy and reliability across diverse orbital conditions, marking a critical advancement for space applications.

### 2.3. Visible-Infrared Image Fusion

Infrared and visible image fusion has been widely researched to combine the thermal radiation information from infrared images with the detailed texture features from visible images, aiming to improve scene perception for downstream vision tasks. Traditional fusion methods can be broadly classified into two categories: multi-scale transformation-based approaches [37] and sparse representation-based methods [38]. Although these methods rely on handcrafted fusion rules, their performance is highly dependent on feature extraction and fusion strategies, which may result in information loss, particularly in complex scenarios.

Recent advances in deep learning have revolutionized this field. Convolutional neural networks (CNNs)-based frameworks, such as IFCNN [39] and FusionGAN [40], leverage hierarchical features to automatically learn fusion rules through adversarial or non-adversarial training. Attention mechanisms further improve feature selection by highlighting salient regions across modalities. AttentionFGAN [41] combined with attention mechanisms and generative adduction networks, significantly improves the visual effect of fused images. Emerging trends focus on hybrid architectures combining deep networks with traditional methods. DenseFuse [42] integrates multi-scale analysis with dense connectivity to enhance feature representation. In addition, research on spacecraft pose estimation based on RGB-Thermal (RGB-T) fusion remains relatively limited. Rondao et al. [43] developed the ChiNet framework, which effectively enhances RGB images by incorporating long-wave infrared data, thereby providing richer feature representations while mitigating the impact of artifacts commonly encountered in visible-light imaging of spatial objects. Notably, Li et al. [44] proposed DCTNet, a heterogeneous dual-branch multi-cascade network for infrared and visible image fusion. DCTNet employs a transformer-based branch and a CNN-based branch to extract global and local features, respectively, followed by a multi-cascade fusion module to integrate complementary information. U2Fusion [45] adapts to various fusion tasks through a single model. However, its generic design does not address the unique challenges of space environments, such as extreme lighting conditions and the need for precise pose estimation. In a complementary study, Hogan et al. [46] demonstrated that thermal imaging sensors offer a viable alternative to conventional visible-light cameras under low-illumination conditions. Their work introduced a novel convolutional neural network architecture for spacecraft relative attitude prediction, achieving promising results. Furthermore, recent advances in Transformer-based models [47] have shown significant potential in capturing long-range dependencies between heterogeneous modalities, with successful applications in RGB-T fusion for visual tracking of non-cooperative space objects.

In this paper, different from the above-mentioned research, we take image fusion as the upstream task of pose estimation and create a noval space non-cooperative object pose estimation framework based on infrared and visible images.

## 3. Methodology

In this section, we provide a novel Visible and Infrared Fused Pose Estimation Framework (VIPE) for space noncooperative objects, and then introduce Fusion subnetwork and Pose estimation network in detail. Finally, the loss function design of the VIPE are presented concretely.

### 3.1. Method Overview

**Figure 1:**
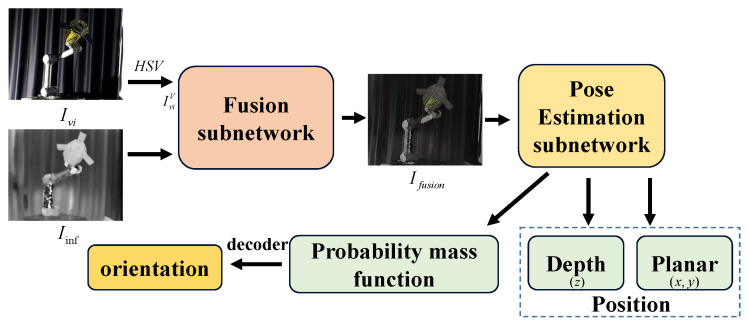
The overall framework of VIPE.

The overall structure of the VIPE is shown in the Figure 1. It can be seen that VIPE mainly consists of two parts, namely fusion subnetwork and pose estimation subnetwork. First, the RGB image is converted to the HSV color channel image, and then the value channel of the visible image and the infrared image are input into the fusion subnetwork to obtain the fused image. The fused image is subsequently used as the input to the pose estimation subnetwork to estimate the position and the pose probability mass function of the non-cooperative object. The position information is composed of plane information and depth information, and the Euler angles around the XYZ axes in the camera coordinate system can be obtained by decoding the pose probability mass function. We will introduce the fusion subnetwork and the pose estimation subnetwork in detail below.

### 3.2. Fusion Subnetwork

**Figure 2:**
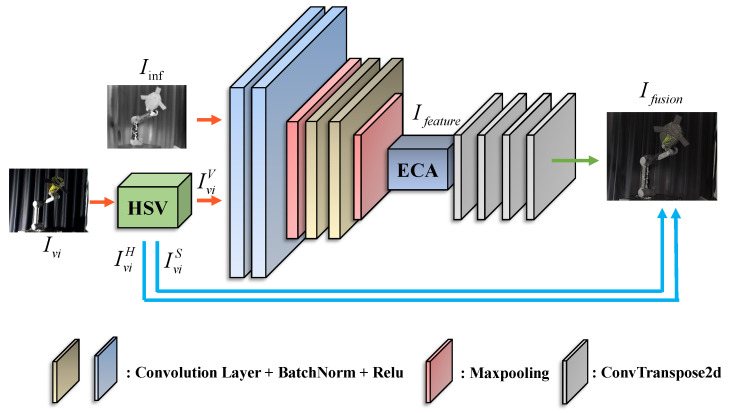
Fusion subnetwork.

In order to realize efficient and robust visible-infrared image fusion, we design an image fusion subnetwork based on the Encoder–Decoder structure. Through multi-level feature extraction and feature enhancement, the network gradually fuses the significant feature information of the non-cooperative object in the input image, and finally generates a high-quality fusion image with the same size as the input image. The structure of fusion subnetwork is shown in Figure 2 and Table 1.

Let $I_{inf}\in R^{H\times W\times 1}$ and $I_{vi}\in R^{H\times W\times 3}$ represent infrared images and visible images of the space non-cooperative object respectively, and input images are assumed to be registered well. As shown in Equation (1), $I_{vi}$ is converted to the HSV color space.

(1) $$I_{vi_{H}},I_{vi_{S}},I_{vi_{V}}=HSV\left(I_{vi}\right)$$

where HSV(·) means to convert the image to RGB color space. $I_{vi_{H}}$,$I_{vi_{S}}$,$I_{vi_{V}}$ represents the Hue, Saturation, Value channel image of the visible image.

In the Encoder, we employ the convolutional layer to represent the input image in an high-dimensional embedding space, thereby obtaining a feature tensor $I_{feature}\in R^{H\times W\times C}$. In Equation (2), *H*, *W*, *C* denote the height, width, and number of channels of the feature tensor, respectively. E(·) denotes the encoder of the fusion subnetwork.

(2) $$I_{feature}=E\left(I_{vi_{V}},I_{inf}\right)$$

**Figure 3:**
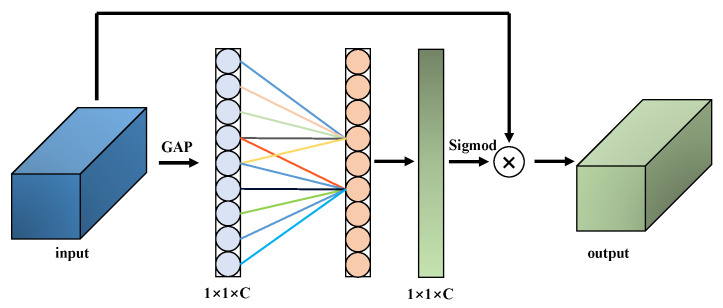
Structure of efficient channel attention.

Through a series of multi-layer convolutional operations, the network is capable of progressively capturing features from low-level to high-level, thereby providing a robust foundation for subsequent feature enhancement and fusion. In order to further improve the ability to express the salient features of the target, we introduced the Efficient Channel Attention (ECA) in the middle layer of the Fusion subnet. The structure of ECA is shown in the Figure 3, ECA can dynamically enhance the significant feature information of the target in the image by weighting the features in the channel dimension.

In the decoder, we design a multi-level decoding structure to restore the feature tensor, of which size is the same as the input image to the fusion image. The kernel size of the first two deconvolution layers of the decoder is 2×2, stride =2, and the convolution kernel of the last two deconvolution layers is 1×1, stride = 1. Specifically, the decoder effectively combines the different levels of feature information in the encoder through Skip Connection.

This design not only preserves the high-resolution details of the input image, but also ensures the integrity and consistency of the feature information to generate a high-quality fusion image, which is then recombined with $I_{vi_{H}}$ and $I_{vi_{S}}$ and converted to RGB image $I_{fusion}$, as shown in Equation (3), which is the input to the pose estimation subnetwork.

(3) $$I_{fusion}=\bar{HSV}\left(D(I_{feature}),I_{vi_{H}},I_{vi_{S}}\right)$$

where $\bar{HSV}\left(\cdot \right)$ represents the conversion of the HSV image to the RGB color space, and D(·) represents the decoder of the fusion subnetwork.

### 3.3. Pose Estimation Subnetwork

**Figure 4:**
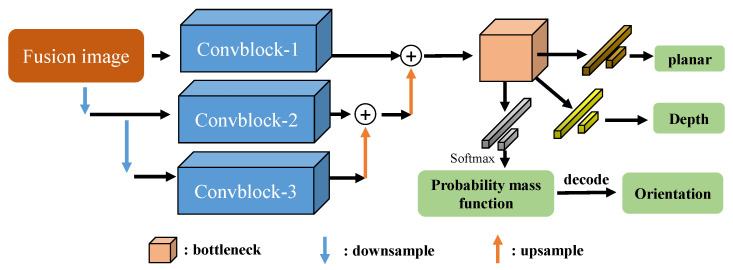
The structure of pose estimation subnetwork.

The pose estimation subnetwork comprises two components: position estimation and orientation estimation. Given the challenges visual cameras face in capturing depth information, position estimation is bifurcated into plane position estimation (XY) and depth position estimation (Z). Pose estimation subnetwork uses an end-to-end regression method to learn the pose mapping relationship of the target from the fusion image, so as to achieve efficient, accurate, and robust pose estimation of space non-cooperative objects. As illustrated in Figure 4, the pose estimation subnetwork consists of three primary stages.

For orientation estimation, we propose a method of soft classification coding to directly get the object’s orientation and encode the label information into a probabilistic mass function in the discrete space as a new orientation label. We first divide Rx, Ry, and Rz of each label into intervals of *M* Gaussian distributions respectively, from which we can obtain the set *T* containing $M^{3}$ discrete space points.

In Equation (4), we designed a kernel function *K* to establish a one-to-one correspondence between the original label information $E_{gt}^{i}=\left(Rx_{gt}^{i},Ry_{gt}^{i},Rz_{gt}^{i}\right)$ and the divided discrete space *T*, and then carried out normalization processing to obtain the label truth value (probability mass function) of the branch of the pose estimation network, as shown in the Equation (5).

(4) $$K\left(E_{gt}^{i},T\right)=e^{-\frac{cos(E_{gt}^{i}-T)}{\mu M^{2}}}$$

(5) $$C\left(E_{gt}^{i},T\right)=\frac{K(E_{gt}^{i},T)}{\sum_{i=1}^{M^{3}}K(E_{gt}^{i},T)}$$

where μ represents the smooth term, *i* represents the number of image pairs in the dataset, and *M* represents the number of intervals of the divided Gaussian distribution. When the pose estimation network is tested, the real non-cooperative object orientation can be obtained by using the probability mass function $\omega_{k}=\left\{\omega_{1},\omega_{2},\omega_{3}...\omega_{M^{3}}\right\}$ of the branch output of the pose estimation network and decoding according to Equation (6).

(6) $$\overset{\wedge }{e}=\underset{e}{\arg\min}\sum_{k}^{M^{3}}\omega_{k}\left(1-\cos\left(e-T\right)\right)$$

#### 3.3.1. Feature Extraction Network

**Figure 5:**
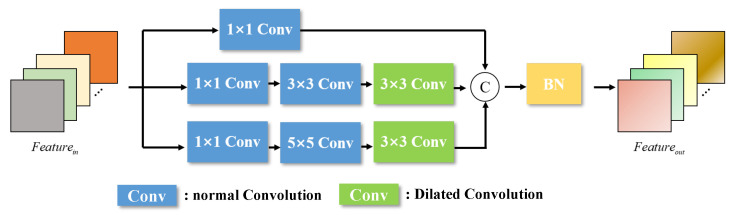
The structure of Convblock.

The overall structure of the feature extraction network is shown in Figure 4. It can be seen that we extracted multi-scale features of the fusion image through multiple down-sampling and up-sampling operations. Given that the pose estimation of non-cooperative objects necessitates the network to learn robust spatial position extraction capabilities, our ConvBlock is designed with multi-scale convolutions. The ConvBlock consists of three branches, as shown in Figure 5.

The first branch primarily includes a convolutional layer with a 1 × 1 convolution kernel, responsible for extracting low-level features. The second branch comprises three convolutional layers with convolution kernels of 1 × 1, 3 × 3, and 3 × 3, respectively. Notably, we employ dilated convolution [48] in the last layer to expand the receptive field and capture a broader range of spatial information. In the third branch, we utilize convolutional layers with convolution kernels of 1 × 1, 5 × 5, and 3 × 3, respectively, and the last layer is also a dilated convolution. Subsequently, by concatenating the feature vectors output from the three multi-scale convolution branches and applying BatchNormalization, we achieve a richer and more robust feature representation.

#### 3.3.2. Pose Estimation Predicts Branch Networks

After passing through a bottleneck network, we use another bottleneck network to extract the feature module into a 1-dimensional flattened 2D feature map, and input the 1-dimensional vector into three different branches for regressing planar position information, depth position information, and orientation probability mass function. The planar position estimation branch consists of two Fully Connected Layers to achieve regression from high-dimensional feature vectors to 2D planar position information.

The depth position estimation branch also contains two fully connected layers, which perform the regression from high-dimensional feature vectors to 1D depth position information. The orientation estimation branch outputs the predicted orientation probability mass function, transforming the high-dimensional feature vector into a probability distribution in the discrete orientation space through multiple fully connected layers and appropriate activation functions. Table 2 shows the pose estimation subnetwork regression networks specifications of VIPE.

#### 3.3.3. Decoding

The orientation branch is implemented using soft assignment coding and outputs Euler Angle orientation information. The core idea is to encode the Euler Angles and convert them into a probability mass function in the discrete orientation space. Finally, the estimated pose can be obtained by decoding the network output containing the probability mass function. We have included pseudocode to clearly outline the implementation process of VIPE in Algorithm 1.
**Algorithm 1** Visible and infrared fused pose estimation (VIPE) **Require:**Infrared image $I_{inf}\in R^{H\times W\times 1}$, Visible image $I_{vi}\in R^{H\times W\times 3}$ **Ensure:**Position (X,Y,Z), Orientation (Rx,Ry,Rz) 1:**Step 1: Image Conversion** 2:Convert $I_{vi}$ to HSV color space: $\left(I_{vi_{H}},I_{vi_{S}},I_{vi_{V}}\right)=HSV\left(I_{vi}\right)$ 3:**Step 2: Fusion Subnetwork** 4:Encode $I_{vi_{V}}$ and $I_{inf}$: $I_{feature}=E\left(I_{vi_{V}},I_{inf}\right)$ 5:Decode $I_{feature}$: $I_{fusion}=\bar{HSV}\left(D\left(I_{feature}\right),I_{vi_{H}},I_{vi_{S}}\right)$ 6:**Step 3: Pose Estimation Subnetwork** 7:Estimate plane position (X,Y) and depth *Z* from $I_{fusion}$ 8:Estimate orientation (Rx,Ry,Rz) using soft classification: 9:   Divide Rx,Ry,Rz into *M* Gaussian intervals to form discrete space *T* 10: Compute probability mass function: $C\left(E_{gt}^{i},T\right)=\frac{K(E_{gt}^{i},T)}{\sum_{i=1}^{M^{3}}K\left(E_{gt}^{i},T\right)}$ 11: Decode orientation: $\hat{e}=\arg\min_{e}\sum_{k=1}^{M^{3}}\omega_{k}\left(1-\cos\left(e-T\right)\right)$ 12:**Output:** Position (X,Y,Z), Orientation (Rx,Ry,Rz)

### 3.4. Loss Function

The VIPE proposed in this work consists of image fusion subnetwork and pose estimation subnetwork. In order to obtain distinctive images and high-precision pose during training, the loss function $L_{total}$ is shown in the Equation (7).

(7) $$L_{total}=\left(L_{fusion},L_{pose}\right)$$

where $L_{fusion}$ represents the fusion loss function, and $L_{fusion}$ includes structure loss function $L_{structure}$ and content loss function $L_{content}$. The specific expressions of $L_{structure}$ and $L_{content}$ are shown in Equations (9) and (11). We hope that the fused image can retain the detailed structure information of the visible image and the intensity information of the infrared image, as shown in Equation (12).

(8) $$L_{fusion}=L_{content}+L_{structure}$$

(9) $$L_{structure}=1-SSIM\left(I_{fusion},I_{vi}\right)$$

(10) $$SSIM\left(x,y\right)=\frac{\left(2\mu_{x}\mu_{y}+C_{1}\right)\left(2\sigma_{xy}+C_{2}\right)}{\left(\mu_{x}^{2}+\mu_{y}^{2}+C_{1}\right)\left(\sigma_{x}^{2}+\sigma_{y}^{2}+C_{2}\right)}$$

where $\mu_{x}$ and $\mu_{y}$ are the local means of images *x* and *y*, $\sigma_{x}^{2}$ and $\sigma_{y}^{2}$ are the local variances, $\sigma_{xy}$ is the local covariance, and $C_{1}$ and $C_{2}$ are constants to stabilize the division.

(11) $$L_{content}=\frac{1}{H\times W}\left|\right|I_{fusion}-\max\left(I_{vi},I_{\inf}\right)\left|\right|$$

The content loss $L_{content}$ measures the pixel-wise intensity difference between the fused image and the maximum intensity values from the visible and infrared images, ensuring that the fused image preserves the salient thermal and texture features.

In Equation (7), $L_{pose}$ contains loss functions of three network branches, where $L_{planar}$, $L_{depth}$, and $L_{probability}$ represent plane position regression loss, depth position regression loss, and orientation soft classification loss respectively.

(12) $$L_{pose}=L_{planar}+L_{depth}+\xi L_{probability}$$

We use mean square error (MSE) to measure the difference between the predicted plane position information and the real plane position information. Similarly, MSE is used to measure the difference between the predicted depth position information and the true depth position information. For the orientation estimation branch, instead of using $L_{planar}$ and $L_{depth}$, we use Cross Entropy losses to train the model, measuring the difference between the predicted orientation and the true orientation. A balancing term ξ is used to adjust the orders of magnitude between the different losses.

## 4. Experimental Results and Analysis

In this section, we present the BVPE dataset and provide comprehensive details on the experimental setup. To evaluate the performance of VIPE, we compared it with other state-of-the-art monocular pose estimation methods on the BVPE dataset and performed an in-depth analysis of the results. Furthermore, we conducted a series of ablation studies to validate the reliability and effectiveness of the proposed image fusion approach.

### 4.1. Dataset

To address the significant challenge of the lack of a space non-cooperative object pose estimation dataset containing both infrared and visible images, we developed BVPE, the first dataset of its kind. BVPE includes spacecraft models exhibiting a wide range of attitude variations, providing a comprehensive resource for pose estimation research.

**Figure 6:**
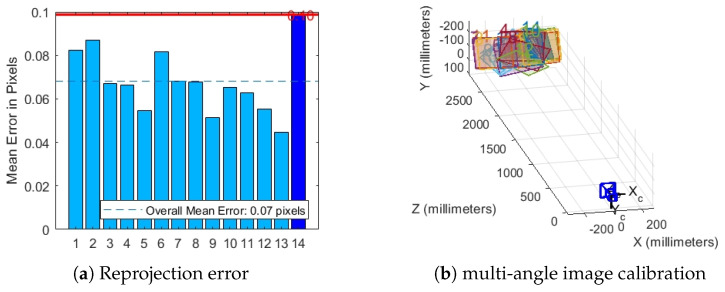
H30T pod internal parameters calibration.

First, we utilize a standard checkerboard to calibrate the camera and derive its intrinsic parameters, including focal length, principal point coordinates, and distortion coefficients. This calibration process corrects various distortions inherent in the camera imaging system, thereby enhancing the accuracy of subsequent image processing. The intrinsic parameters of the camera are provided in Equation (13). As illustrated in Figure 6, we present the reprojection error and multi-angle image calibration results obtained during the intrinsic parameter calibration process.

(13) $$M_{intr}=\left[\begin{array}{ccc} 1764.245 & 0 & 975.3085\\ 0 & 1762.718 & 531.5116\\ 0 & 0 & 1 \end{array}\right]$$

Then, we used the UR5 robotic to acquire images of the calibration plate from different positions and orientations, and obtained the base coordinate transformation matrix between the camera coordinate system and the mechanical arm through hand-eye calibration, as shown in Equation (14). The true pose value of the non-cooperative object in the camera coordinate system is obtained using the UR5 robot teaching device.

(14) $$M_{ext}=\left[\begin{array}{cccc} 0.9983 & -0.0101 & -0.0559 & 112.6244\\ 0.0559 & 0.0068 & 0.9984 & -2919.4689\\ -0.0097 & -0.9999 & 0.0073 & 580.2182\\ 0 & 0 & 0 & 1 \end{array}\right]$$

We used the H30T pod to capture images of the spacecraft model from various angles and distances. The specific parameters of the H30T pod are listed in Table 3, which includes both the visible sensor and the infrared sensor. By adjusting the RTSP protocol of the H30T, bimodal image pairs can be obtained. We designed a multi-threaded program to capture visible light and infrared images at the same time as much as possible using the H30T. Subsequently, the spatial registration of visible-infrared image pairs was achieved based on the ORB algorithm.

**Figure 7:**
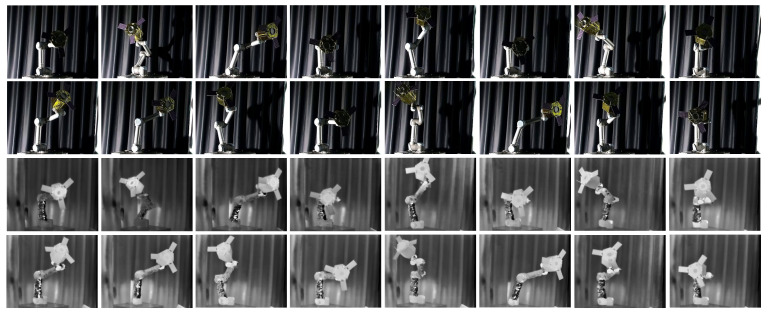
BVPE dataset. The first two rows are visible images and the second two rows are infrared images after registration.

We ensured that the BVPE dataset included samples from various pose changes and environmental conditions to enhance the model’s generalization. To address the resolution disparity between visible (1920 × 1080) and infrared (640 × 512) images, we utilized the ORB algorithm to spatially align the raw images, ensuring a consistent resolution of 640 × 512 for both modalities. Using high-precision measurement equipment and calibration results, we obtained the real pose parameters (including the X and Y axes for planar position, the Z axis for depth position, and the azimuth Euler angle) of each sample in the camera coordinate system. These ground truth data are used to train and evaluate the model’s attitude estimation performance. Ultimately, the BVPE dataset comprised 3630 pairs of spatially aligned visible and infrared images, in which 2900 pairs are used for training and the remaining 730 pairs are used for testing. Ultimately, the BVPE dataset comprised 3630 pairs of images, in which 2900 pairs are used for training and the rest are used for testing, and we show part of the BVPE dataset in Figure 7.

**Figure 8:**
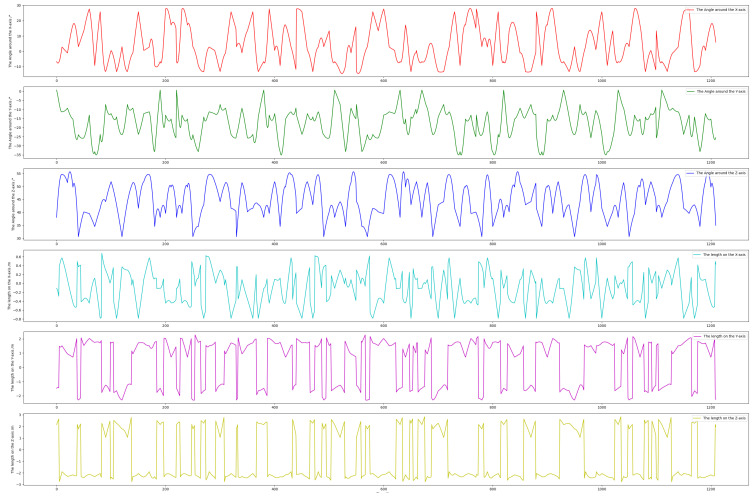
The pose transformation state of the effector of the UR5 robotic arm during constructing the BVPE dataset.

To comprehensively capture diverse satellite poses for our BVPE dataset, we precisely controlled a UR5 robotic arm to manipulate the satellite model through wide-ranging rotational motions while carefully maintaining the arm inside the camera’s field of view and avoid visual occlusion(with an orientation feedback accuracy of 0.01°). As shown in Figure 8, the first three subplots depict the end-effector’s angular variations around the X, Y, and Z axes (each reaching approximately 70°), while the remaining subplots display the corresponding translational displacements in the base coordinate system (all exceeding 2 m). Figure 8 provides a detailed visualization of the UR5 robotic arm’s pose transformation state during the dataset construction process. It quantifies the angular and translational variations of the end-effector, which directly correspond to the poses captured in Figure 7. These substantial motion ranges (including rotation and translation) indicate that BVPE encompasses extensive and representative satellite attitude distributions, and diversity in pose coverage ensures the robustness of pose estimation for non-cooperative space objects.

### 4.2. Implementation Details and Metrics

In the process of network training, we first train the image fusion sub-network separately to ensure that the quality of the generated fusion images is optimal. After this training is completed, we freeze the parameters of the image fusion sub-network and then continue to train the pose estimation sub-network. This allows the pose estimation sub-network to learn from high-quality fusion images and accurately estimate the pose information of the target. Through two-stage training strategy, we can not only gradually optimize the performance of each sub-network but also effectively reduce the computational burden during training, thereby improving the training efficiency of the whole model and the final pose estimation accuracy.

As for the training of fusion subnet, the input image size is 640×512. The optimizer is selected as Adam with a learning rate of 1 × 10^−3^. The batch size =10 and the epoch =100. The learning rate decays to 80% of its original value every 7 epochs. As for the training of pose estimation subnet, lr = 1 × 10^−4^, batch size = 5, using the Adam optimizer for training, ξ=10. The networks proposed in this paper are all based on Pytorch, a deep learning framework, trained on Tesla P100 GPUs and a 2.60 GHz Intel(R) Xeon(R) Gold 6132.

The evaluation metrics we adopted are inspired by the European Space Agency (ESA) standards but tailored to our specific requirements. While ESA primarily uses quaternion-based orientation representation, our labels are based on Euler angles, and thus our evaluation metrics include $E_{pos}$ and $E_{ori}$. For position estimation, we calculate the L2-norm between the predicted position (*X*, *Y*, *Z* axis) and the true position as $E_{pos}$, as shown in Equation (15). For attitude estimation, we introduce a new metric, $E_{ori}$, which computes the absolute value of the difference between the predicted and true orientation Euler angles, as shown in Equation (16).

(15) $$E_{ori}=\frac{1}{N}\sum_{i}^{N}(\sqrt{{\left(Rx_{gt}^{i}-Rx_{predict}^{i}\right)}^{2}+{\left(Ry_{gt}^{i}-Ry_{predict}^{i}\right)}^{2}+{\left(Rz_{gt}^{i}-Rz_{predict}^{i}\right)}^{2}})$$

(16) $$E_{pos}=\frac{1}{N}\sum_{i}^{N}(\sqrt{{\left(X_{gt}^{i}-X_{predict}^{i}\right)}^{2}+{\left(Y_{gt}^{i}-Y_{predict}^{i}\right)}^{2}+{\left(Z_{gt}^{i}-Z_{predict}^{i}\right)}^{2}})$$

where *N* represents the number of images, $X_{gt}^{i}$, $Y_{gt}^{i}$, $Z_{gt}^{i}$ and $X_{predict}^{i}$, $Y_{predict}^{i}$, $Z_{predict}^{i}$ represent the ground truth and predicted values of non-cooperative object position along the XYZ axes in the camera coordinate system for the i-th image, respectively. $Rx_{gt}^{i}$, $Ry_{gt}^{i}$, $Rz_{gt}^{i}$ and $Rx_{predict}^{i}$, $Ry_{predict}^{i}$, $Rz_{predict}^{i}$ represent the ground truth and predicted values of the orientation around the XYZ axes for the i-th image, respectively.

### 4.3. Experimental Result and Analysis

#### 4.3.1. Image Fusion

**Figure 9:**
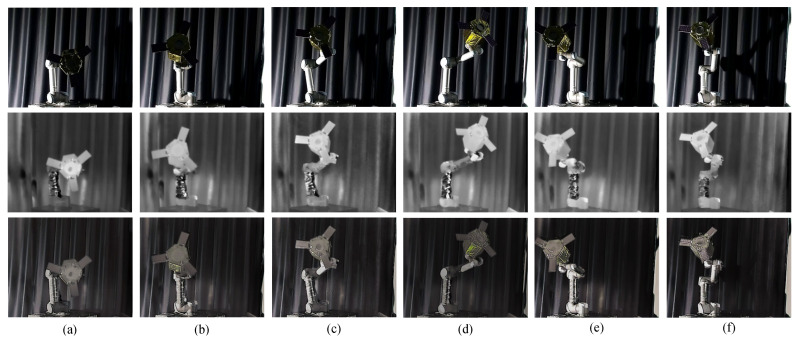
Fusion result. The first row is the visible image, the second row is the infrared image, and the third row is the fused image. (**a**–**f**) represent the image fusion results for six different poses, respectively.

The experimental results of the image fusion sub-network are shown in the Figure 9. We present the visible, infrared, and fused images of six groups of space non-cooperative objects in different poses. As shown in Figure 9, the infrared images can still maintain the intensity of the non-cooperative objects under harsh lighting conditions, especially in Figure 9a–c, where it can be seen that the infrared images effectively supplement the content of the satellite wings and outline in the visible images. Additionally, it can be observed in Figure 9d–f that the color information of the satellite contained in the visible images is retained when the lighting conditions improve. Therefore, we can subjectively conclude that the fused images effectively combine the advantages of visible and infrared images while retaining the details of the object, thereby enhancing the overall information content and clarity of the images.

In addition, we conducted a quantitative analysis of the visible image, infrared image and fusion image, and selected several evaluation metrics, namely entropy (EN) [49], average gradient (AG), standard deviation (SD) [50], spatial frequency (SF), and qabf [51] to evaluate the quality of the images.

**Figure 10:**
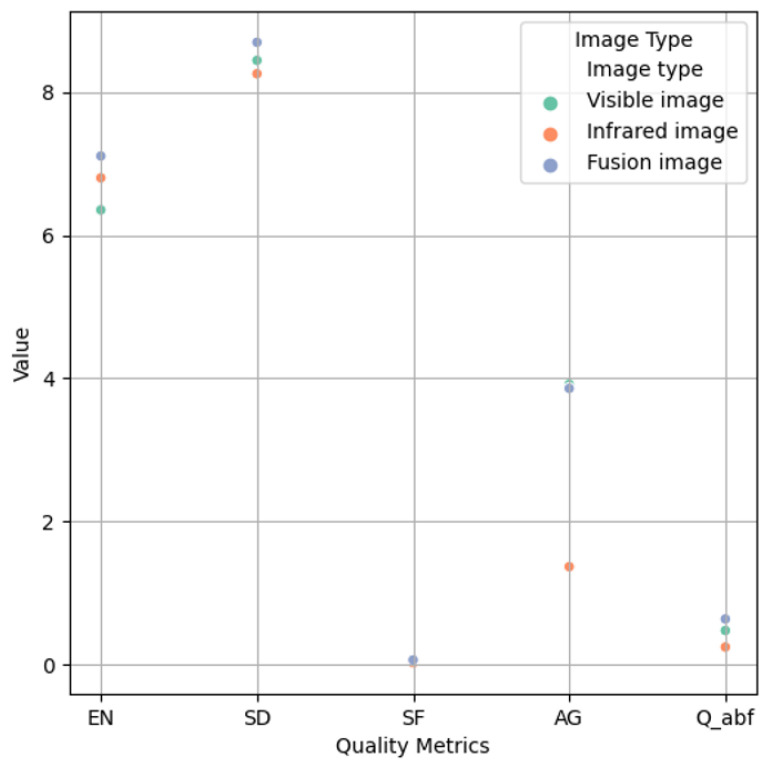
Scatter plot of quality metrics.

The qualitative experimental results are shown in Table 4 and Figure 10. We present the average results of 30 groups of fusion experiments. EN is a measure of the amount of image information. AG reflects the richness of edges and details in the image. SD measures the contrast of the image. SF reflects the content of high-frequency components in the image. QABF is an image quality evaluation metric based on phase consistency. The higher the value of the above five evaluation metrics, the better the quality of the image. It can be concluded that the fused images improve the quality of the images both subjectively and objectively, providing a guarantee for our follow-up high-precision pose estimation.

**Figure 11:**
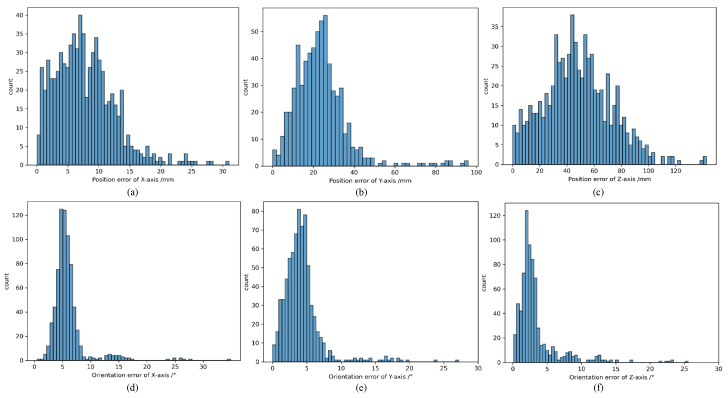
Orientation error distribution and position error distribution of VIPE on BVPE dataset. (**a**–**c**) represent the distribution of position estimation errors, while (**d**–**f**) represent the distribution of orientation estimation errors.

The results of error distribution are shown in Figure 11. It can be seen that the orientation errors around the XYZ axes are primarily concentrated in the range of 0 to 5 degrees, with some images exhibiting larger errors due to occlusion. On the other hand, the position estimation of the non-cooperative object is very accurate. Compared to the X and Y axes in the camera coordinate system, the depth information estimation along the Z axis still has a significant error.

We compared the methods of URSONet, PoseCNN [52], SPN, and SPNv2, the experimental results are shown in Table 5. In the process of comparison, since other methods are based on monocular images, we used the visible image from the BVPE dataset as their input. For VIPE, we respectively used the visible images, infrared images, and fused images as inputs. According to Table 5, we found that the proposed method achieves the best results in both orientation and position estimation of space non-cooperative objects when using the fused image as the input.

#### 4.3.2. Pose Estimation

We calculate the orientation error distributions of non-cooperative objects around the XYZ axes and the position error distributions in the XYZ directions. The error calculations are directly derived from the absolute value of difference between the label true value and the corresponding predicted value, and all experiments are based on 730 pairs of images in the BVPE test set.

**Figure 12:**
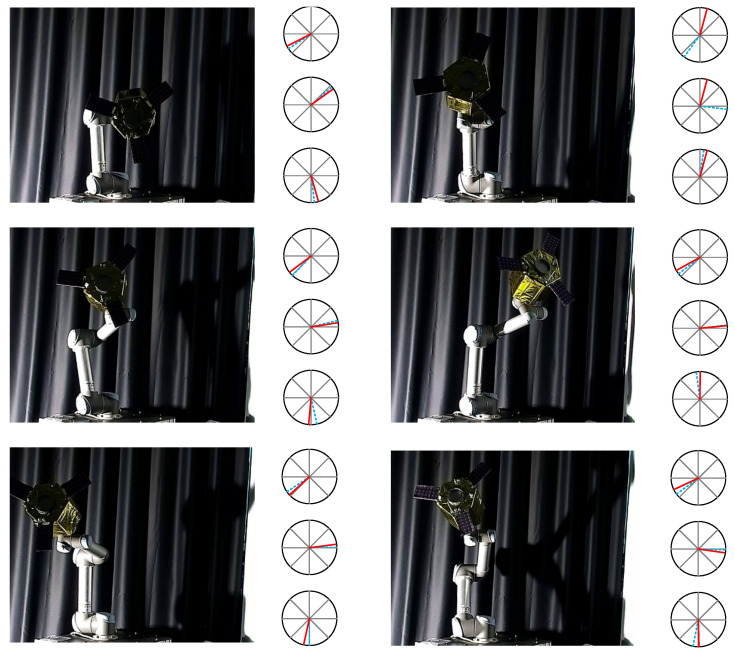
Visualization of pose estimation by visible images (red solid line denotes the pose prediction, and blue dash line denotes the pose ground-truth).

**Figure 13:**
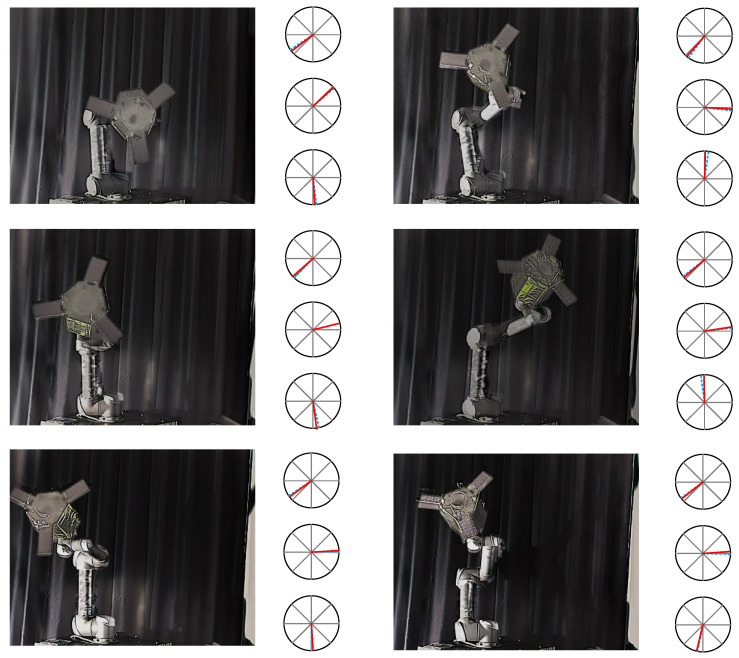
Visualization of pose estimation by fusion images (red solid line denotes the pose prediction, and blue dash line denotes the pose ground-truth).

In addition, we show the visual effects of pose estimation for six groups of images, as illustrated in Figure 12 and Figure 13. Figure 12 displays the experimental results of pose estimation using only visible images, while Figure 13 shows the experimental results of pose estimation using fused images as input. The right portion of each image represents the orientation estimates for the X, Y, and Z axes in polar coordinates, with the dashed blue line indicating the true value and the red line indicating the predicted value.

### 4.4. Ablation Study

**Figure 14:**
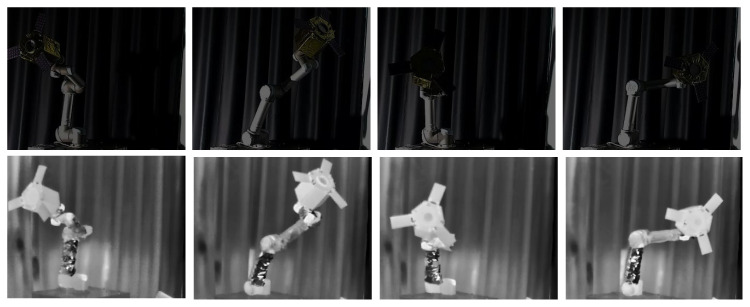
BVPE-Dark dataset.The images in the first row are visible images, while the images in the second row are the corresponding infrared images.

To validate the efficacy of the VIPE structure, we performed comprehensive ablation studies. Notably, to rigorously examine the benefits of fusing infrared and visible-light images for pose estimation of non-cooperative space objects under low-illumination conditions, we specifically developed the BVPE-Dark dataset by substantially reducing ambient lighting intensity during image acquisition. Figure 14 displays representative samples from the BVPE-Dark dataset, which consists of 600 rigorously annotated infrared-visible image pairs acquired under near-darkness conditions.

First, we eliminate the Fusion subnetwork, resulting in the w.o. Fusion subnetwork, where the visible image is directly input into the pose estimation subnetwork. Subsequently, we retain the overall structure of the fusion subnetwork but eliminate the ECA module, denoting it as w.o.Fusion subnetwork-ECA. In the third ablation experiment, we retain only one Convblock in the feature extraction network of the pose estimation subnetwork, thereby eliminating multi-scale sampling, denoting it as w.o. Pose Estimation-MSF. The ablation experiment results on the BVPE dataset and the BVPE-Dark dataset are shown in Table 6. We still use the metrics described in Section 4.2.

From the results, it can be seen that the biggest impact on pose estimation precision is the entire fusion subnetwork. The fusion subnetwork plays a key role in the model by improving pose estimation accuracy through the multiband fusion of information. Eliminating the fusion subnetwork results in the model losing its ability to extract and integrate information from multiple sensors, leading to a significant decline in pose estimation accuracy. The fusion subnetwork of the ECA module is used to enhance channel characteristics and improve the power of feature expression. Although the overall structure of the fusion subnetwork is preserved, the model without the ECA module cannot effectively focus on important channel features during the feature fusion process, resulting in a decrease in pose estimation accuracy. Additionally, eliminating the pose estimation subnetwork also causes a decline in pose estimation precision. Multi-scale feature extraction and sampling in the network are very important, as they can capture feature information at different scales, thereby improving the robustness and accuracy of the model. Retaining only one convolutional block weakens the model’s ability to perform multi-scale feature extraction.

**Figure 15:**
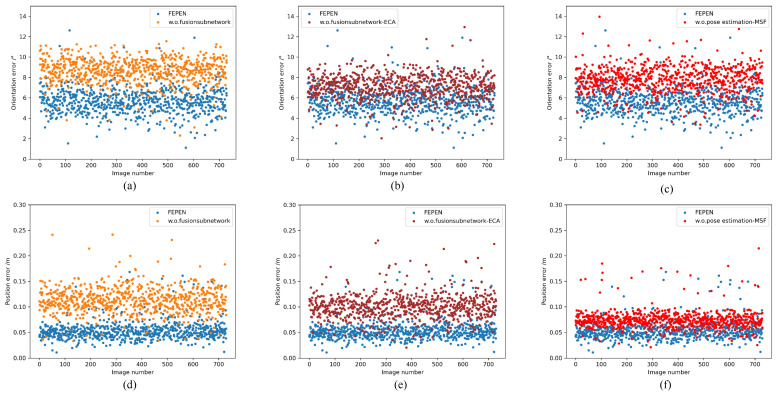
Results of the ablation study. (**a**–**c**) and (**d**–**f**), respectively, show the $E_{ori}$ and $E_{ori}$ on BVPE test sets. Blue represent VIPE, orange represent w.o.fusion subnetwork, brown represent w.o.fusion subnetwork-ECA, and red represent w.o.pose estimation-MSF.

Notably, the ablation study reveals that removing the fusion subnetwork from VIPE severely compromises its performance on the BVPE-Dark dataset, rendering the model unable to extract meaningful pose information or achieve convergence. As demonstrated in Table 6, this finding underscores the critical role of the fusion network in estimating poses of non-cooperative targets under low-illumination conditions. More importantly, these results provide empirical evidence that incorporating infrared imagery effectively compensates for the inherent illumination sensitivity of visible-light images, thereby enhancing the robustness of pose estimation in challenging lighting environments. In addition, in order to visually display the results of the ablation experiment, scatter plot Figure 15 was drawn, where (a), (b), and (c) showed $E_{ori}$ in different states, and (d), (e), and (f) showed $E_{pos}$.

In the pose estimation subnetwork of VIPE proposed in this paper, we conduct orientation estimation based on soft classification. The number of angles divided into different dimensions, *M*, has a significant impact on the experimental results, which determines the dimensionality of the probability mass function output by VIPE. We verify this by setting different value of *M*. The experiments are based on the BVPE dataset’s training and test sets. The experimental results are shown in the Table 7. It can be seen that the accuracy of orientation estimation is the highest when M=9. This is consistent with our expectations, as when *M* is large, the model may have difficulty converging to the optimal value, and when *M* is small, the decoder has difficulty fitting the best posture.

To investigate the impact of the parameter ξ on the performance of attitude estimation, we conducted experiments on the BVPE dataset and recorded the $E_{ori}$ (orientation estimation error) for both the training and testing sets under different values of ξ. The results, as shown in Table 8, indicate that as ξ increases, the $E_{ori}$ initially decreases, reaching its minimum value at ξ=10. However, when ξ is further increased to 50, the $E_{ori}$ rises to 7.0361 (training) and 10.4852 (testing). These findings highlight the importance of carefully tuning ξ to achieve the best performance in attitude estimation tasks.

### 4.5. Robustness Experiment

**Figure 16:**
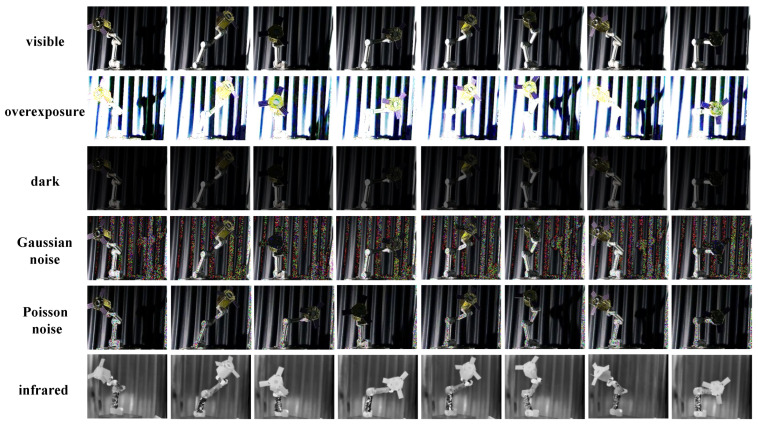
Four perturbations are added to the visible images in the BVPE dataset. From the first row to the last row, they are the original visible images, with overexposure perturbation added, dark environment perturbation, Gaussian noise, Posson noise, and infrared images respectively.

We have added a dedicated chapter focusing on robustness experiments. These experiments play a crucial role in evaluating the performance and reliability of our algorithms under various challenging conditions. In this section, we have incorporated four specific types of perturbations (overexposure, dark, Gaussian noise, Poisson noise) into the space non-cooperative object pose estimation BVPE datasets to comprehensively test the robustness of VIPE, as shown in Figure 16.

By introducing overexposure into the BVPE dataset, we simulate scenarios where the imaging system is exposed to excessive light. Analyzing how our methods perform under overexposure conditions understand their ability to handle extreme lighting situations and maintain accurate pose estimation.

The dark condition in the dataset mimics low-light scenarios that are common in space, like when a target is in the shadow of another object or during the night-side of an orbit. Studying the VIPE’s behavior in the dark environment allows us to assess its sensitivity to limited light and its capacity to extract useful information for pose determination.

Gaussian noise is a type of random noise that often occurs in imaging sensors due to factors such as thermal fluctuations. Poisson noise is related to the statistical nature of photon detection in imaging systems. By including Poisson noise and Gaussian Noise in the dataset, we can examine the VIPE’s robustness against this specific type of noise and its impact on pose estimation accuracy.

In Table 9, we compared the pose estimation accuracies under different perturbations. The experimental results were all obtained by adding different types of perturbations to 730 visible images in the BVPE test set. To demonstrate the effectiveness of VIPE, we conducted tests by inputting only visible images into VIPE and by inputting both visible and infrared images into VIPE simultaneously. The experimental results indicate that when only visible images are input into VIPE, the model can achieve relatively accurate pose estimation under normal lighting conditions and slight perturbations, showing certain performance advantages. However, when faced with complex lighting variations and strong noise interference, the estimation accuracy is affected to a certain extent.

In sharp contrast, when both visible and infrared images are input into VIPE simultaneously, there is a qualitative leap in the model’s pose estimation accuracy. Under extreme lighting conditions such as over-exposure and darkness, the thermal radiation information carried by the infrared images provides additional positioning clues for the model, compensating for the deficiencies of visible images in these scenarios. In the presence of Gaussian noise and Poisson noise interference, the fusion image enables VIPE to filter noise more effectively, enhancing the stability and reliability of features.

Overall, these additional robustness experiments and the inclusion of perturbed datasets enhance the credibility and practical applicability of our research, providing a more comprehensive evaluation of the VIPE.

## 5. Conclusions

In this study, we propose a novel Visible and Infrared Fused Pose Estimation Framework (VIPE) for accurate pose estimation of space non-cooperative objects. VIPE combines a self-supervised image fusion subnetwork and a pose estimation subnetwork to extract and fuse multi-scale features from both visible and infrared images, effectively preserving the detailed texture of visible images and the intensity information of infrared images. To validate our approach, we introduce the Bimodal-Vision Pose Estimation (BVPE) dataset, the first of its kind, which includes diverse spacecraft models with varying poses. Extensive experiments on the BVPE dataset demonstrate that VIPE significantly outperforms traditional pose estimation methods relying on single-modality images. In addition, in this paper, ablation experiments were conducted to verify the effectiveness of each component of VIPE. Meanwhile, four kinds of space perturbations were introduced to verify the robustness of VIPE under different image degradation conditions. VIPE’s robustness in diverse and low-visibility scenarios highlights its potential for applications such as satellite servicing, space debris management, and on-orbit assembly.

Future work will focus on further optimizing the network architecture and exploring the integration of additional sensor modalities to enhance the system’s performance and adaptability. At the same time, it will develop visible and infrared image pose estimation datasets suitable for the pose estimation method combining key point detection and PnP solver. Furthermore, we will acquire advanced equipment to simulate space-like conditions, including gravitational effects and atmospheric absence, to better validate the generalization and robustness of our framework in real-world scenarios. These efforts will significantly contribute to the advancement of pose estimation techniques for space applications.

## Figures and Tables

**Table 1:** VIPE image fusion subnetwork.

Layer	Kernel Size	Stride	Channels
Convolution Layer 1	3 × 3	1	64
Convolution Layer 2	3 × 3	1	256
Pooling Layer 1	-	2	256
Convolution Layer 3	3 × 3	1	256
Convolution Layer 4	3 × 3	1	512
Pooling Layer 2	-	2	512
ECA Layer	-	-	512
Transposed Convolution Layer 1	2 × 2	2	256
Transposed Convolution Layer 2	2 × 2	2	128
Transposed Convolution Layer 3	1 × 1	1	32
Transposed Convolution Layer 4	1 × 1	1	1

**Table 2:** Pose estimation subnetwork regression networks specifications.

Network	Layer	Input	Output	Dropout
Planar Position	Fully Connected Layer 1	1024	256	0.5
Planar Position	Fully Connected Layer 2	256	64	0.5
Planar Position	Fully Connected Layer 3	64	2	-
Depth Position	Fully Connected Layer 1	1024	256	0.5
Depth Position	Fully Connected Layer 2	256	64	0.5
Depth Position	Fully Connected Layer 3	64	1	-
Probabilistic Mass Function	Fully Connected Layer 1	1024	256	0.5
Probabilistic Mass Function	Fully Connected Layer 2	512	256	0.5
Probabilistic Mass Function	Fully Connected Layer 3	256	64	-

**Table 3:** The specific parameters of HT30.

Paramaeter	Visible Sensor	Infrared Sensor
Resolution ratio	1920 × 1080	640 × 512
Focus Distance	15 mm	19 mm
Horizontal field Angle	58.1°	22.9°
Frame rate (IB)	50 fps	15 pfs
Spectral range	400–760 nm	8–14 μm

**Table 4:** The average of five quality metrics for 30 groups images from the BVPE dataset. **BOLD** indicates the best result.

Image Type	EN	SD	SF	AG	$Q_{\mathrm{abf}}$
Visible image	6.3537	8.4443	0.0433	**3.9171**	0.4737
Infrared image	6.8023	8.2575	0.0213	1.3663	0.2422
Fusion image	**7.1075**	**8.6973**	**0.0623**	3.8582	**0.6342**

**Table 5:** Comparison between the proposed method and different single stage methods.

Method	Visible Image	Infrared Image	$E_{\mathrm{pos}}$	$E_{\mathrm{ori}}$
URSONet	✓	×	8.3047	0.084
PoseCNN	✓	×	8.5802	0.174
SPN	✓	×	7.0158	0.139
SPNv2	✓	×	6.4453	0.077
	✓	×	8.8456	0.113
VIPE	×	✓	7.7981	0.440
	✓	✓	**5.6810**	**0.052**

**Table 6:** Ablation study results.

Methods	BVPE Dataset		BVPE-Dark Dataset	
	$E_{\mathrm{ori}}$	$E_{\mathrm{pos}}$	$E_{\mathrm{ori}}$	$E_{\mathrm{pos}}$
w.o.fusion subnetwork	8.8456	0.113	48.5177	0.457
w.o.fusion subnetwork-ECA	7.2208	0.101	9.5517	0.198
w.o.pose estimation-MSF	7.9721	0.074	12.7004	0.122
VIPE	**5.6810**	**0.052**	**9.3421**	**0.067**

**Table 7:** Performance comparison of orientation soft classification parameters on BVPE and BVPE-Dark datasets.

*M*	BVPE Dataset		BVPE-Dark Dataset	
	$E_{\mathrm{ori}}$ **(Train)**	$E_{\mathrm{ori}}$ **(Test)**	$E_{\mathrm{ori}}$ **(Train)**	$E_{\mathrm{ori}}$ **(Test)**
4	13.5309	21.7032	18.8921	24.5612
8	8.2201	13.4840	9.7654	15.2309
9	**3.1529**	**5.6810**	**5.8735**	**9.3421**
12	5.2318	9.3902	8.1526	12.8923

**Table 8:** Performance comparison of ξ on BVPE dataset.

ξ	$E_{\mathrm{ori}}$ (Train)	$E_{\mathrm{ori}}$ (Test)
0.1	42.5541	45.1409
1	14.0787	19.4570
10	**3.1529**	**5.6810**
50	7.0361	10.4852

**Table 9:** Robustness experiment results.

Input	BVPE		Overexposure		Dark		Gaussian Noise		Poisson Noise	
	$E_{\mathrm{ori}}$	$E_{\mathrm{pos}}$	$E_{\mathrm{ori}}$	$E_{\mathrm{pos}}$	$E_{\mathrm{ori}}$	$E_{\mathrm{pos}}$	$E_{\mathrm{ori}}$	$E_{\mathrm{pos}}$	$E_{\mathrm{ori}}$	$E_{\mathrm{pos}}$
Visible	8.8456	0.113	22.4785	0.136	48.5177	0.457	11.6307	0.210	10.3790	0.187
Visible & Infrared	7.2208	0.101	7.681	0.097	9.5517	0.198	9.367	0.168	7.489	0.127

## Data Availability

The original contributions presented in this study are included in the article. Further inquiries can be directed to the corresponding author.
